# The impact of nationality status and place of residency on health-related social needs and quality of life among Palestinian Refugees in Jordan

**DOI:** 10.1016/j.heliyon.2025.e42552

**Published:** 2025-02-08

**Authors:** Hamza Alduraidi, Rahaf Issam Abu Zayid, Noor Mahmoud Jaber, Bassam Saleh Hijazi, Sa'ed Radwan Khamis, Hasan Fahed Awad, Marah Al-Khateeb, Saif alnour Mahmoud Aldhirat, Ahmad Amjed Toubasi

**Affiliations:** a School of Nursing, The University of Jordan, Amman 11942, Jordan; b School of Medicine, The University of Jordan, Amman 11942, Jordan; c School of Medicine, Jordan University of Science and Technology, Irbid 22110, Jordan; d School of Medicine, Mutah University, Kerak 61710, Jordan

**Keywords:** Health-related social needs, Health-related quality of life, Palestinian refugees, Camp residency, Citizenship, Jordan

## Abstract

**Introduction:**

Large number of Palestinian refugees reside in Jordan; most of whom enjoy Jordanian citizenship, and most of whom reside outside camps. Poverty, unemployment, and a poor health profile are more prevalent among camp-residing refugees and non-citizen refugees. This study aims to assess differences in health-related social needs and quality of life among different Palestinian refugee communities in Jordan, based on their nationality status and camp residency.

**Methods:**

A descriptive, comparative, cross-sectional design was used to measure health-related social needs (using the Accountable Health Communities Health-related Social Needs (AHCHRSN) screening tool) and quality of life (using the World Health Organization Quality of Life BREF (WHOQOL-BREF)) among a sample of 236 adult Palestinian refugees in three contexts; (off-camp, in-camp citizens, and in-camp non-citizens) in Jordan.

**Results:**

All participants had needs in the mental health domain, and 179 (75.8 %) suffered from food insecurity. Living in Gaza and Husn camps correlated with increased odds of safety needs (OR = 4.27, 95%CI: 1.541 to 11.76 and OR = 2.936, 95%CI: 1.942 to 9.147) and financial needs (OR = 6.135, 95%CI: 2.062 to 18.181 and OR = 3.932, 95%CI: 1.765 to 8.210) compared to living outside camps. Gaza and Husn camps were associated with higher odds of employment needs (OR = 2.349, 95%CI: 1.877 to 6.291 and OR = 2.406, 95%CI: 1.050 to 5.514) compared to living outside camps. Quality of life scores did not vary significantly between participants in the three settings.

**Conclusion:**

Health-related quality of life scores are generally low across all Palestinian refugee communities. A Palestinian refugee who lives in Gaza camp is four times more likely to have safety needs, six times more likely to have financial needs, and over twice more likely to have employment needs than a refugee outside camps. Despite having citizenship, a Palestinian refugee who lives in Husn camp is three times more likely to have safety needs, four times more likely to have financial needs, and over twice more likely to have employment needs than a refugee outside camps. International states and organizations should help meet these needs by funding the United Nations’ Relief and Work Agency for Palestinian Refugees in the Near East (UNRWA) and the Jordanian government. Further research is needed to understand the lived experience of Palestinian refugees, and the impact of lacking citizenship and residing in camps on their lives and livelihoods, especially after *the coronavirus disease 2019 (COVID-19*) pandemic.

## Highlights

• Living within refugee camps without citizenship is linked to greater economic hardship and worse living conditions.
• All Palestinian refugees suffer from poor mental health.
• Camp residents have higher safety, educational, and employment needs.
• Limited data exist on Palestinian refugee residents.

## 1 Introduction

Throughout history, armed conflicts have consistently stood as one of the most profound catastrophes challenging humanity. The aftermath of warfare has given rise to a multitude of issues, spanning from loss of life, injuries, and mass migrations, to global, financial, and environmental challenges. A statistical approximation reveals that 2640 million people were killed in the last 5500 years due to warfare [1]. Moreover, an estimated 281 million people, 3.6 % of the world's population, currently reside outside their homeland [2].

In May 1948, the commencement of the first Arab-Israeli conflict took place, resulting in the displacement of approximately 750 thousand Palestinians from their land and seeking safe refuge in neighboring Arab states, including Jordan. In the early 1950's, the Jordanian authorities issued a law that granted Jordanian citizenship to Palestinian refugees who fled to Jordan and resided in it – mostly in refugee camps- and their descendants, because the West Bank and East Jerusalem territories were under the Jordanian administration at that time [3]. In June 1967, the second Arab-Israeli war took place, resulting in the Israeli annexation and occupation of the Palestinian territories of West Bank and Gaza strip, causing a second major wave of Palestinians refugees in Jordan. The second wave of Palestinian refugees in Jordan -mostly Gazans-did not enjoy full Jordanian citizenship though, because of the different socio-political circumstances in the region in late 1960's [4].

According to the United Nations' Relief and Work Agency for Palestinian Refugees in the Near East (UNRWA), a Palestinian refugee is defined as “a person whose normal place of residence was Palestine during the period June 1, 1946 to May 15, 1948, and who lost both home and means of livelihood as a result of the 1948 conflict,” and their descendants [4], regardless of their citizenship status. Currently, the global count of Palestinian refugees has surged to 5.9 million individuals [5]. Jordan, a lower middle-income country with a population of over 11 million, is the foremost and most extensive host country for Palestinian refugees worldwide. This is underscored by the fact that roughly 40 % of the Jordanian population are of Palestinian descent, but are not necessarily registered as refugees, as documented by UNRWA in 2016. Jordan currently provides refuge to over two million officially registered Palestinian refugees, as it has recorded thirteen Palestinian refugee camps; ten of which are officially registered by UNRWA such as Gaza and Husn camps. Those registered camps host around 17.4 % of Palestinian refugees, while the remaining 82.6 % reside either in the three unregistered camps, or outside camps among urban population in Jordan [6]. While Jordan is thriving in most of the United Nations' 17 sustainable developmental goals [SDG's], gaps still exist in certain demographics in the Jordanian populations, including refugee communities. The first SDG is ending poverty, and the eighth SDG is ensuring decent work to those who seek it. However, there is a clear concentration of poverty and unemployment among Palestinian refugees inside camps in Jordan, as compared to refugees outside camps, or to Jordan's general population [7].

Gaza camp, an officially-registered UNRWA Palestinian refugee camp, also known as Jerash camp, was established in 1968 to accommodate 11,500 Palestinian refugees who were displaced from the occupied Gaza Strip in the 1967 s Arab-Israeli war. is located 52 km to the north of the capital of Jordan, Amman. Unlike most Palestinian refugees in Jordan who reside in camps established in the early 1950's, the residents of Gaza camp do not enjoy full Jordanian citizenship. As documented in the 2013 Fafo report, Gaza camp represents the poorest among such settlements in Jordan, with more than half of its residents living below the national poverty line. Furthermore, 88 % of its population lack health insurance [8]. This situation primarily originates from the non-citizenship status of a significant portion of Gaza camp's population, which has resulted in substantial disparities in civil rights protection and living conditions [9]. Moreover, accessing and enrollment in education has long been a source of concern within Gaza camp, with approximately 50 % of children aged 5–18 not enrolled in formal educational institutions, and 26 % are not enrolled in formal nor non-formal education programs. Additionally, there has been a noticeable decline in enrollment among adolescents and young adults, as 43%of those between the ages of 15–24 are not participating in any kind of formal educational programs [10].

In contrast, Husn camp, an officially-registered UNRWA Palestinian refugee camp which houses Palestinian refugees displaced from the West Bank and East Jerusalem, is located 80 km north of Amman. Therefore, most of Husn camp inhabitants possess full Jordanian citizenship. Nevertheless, 23 % of Husn camp refugees fall below the national poverty line, and nearly half of them lack access to health insurance. It has also registered the highest unemployment rate among other Palestinian refugee camps in Jordan with 18 % and a 25 % unemployment rate among females [11].

Most Palestinian refugees in Jordan reside outside camps. In Amman, for example, 57 % of households are UNRWA-registered Palestinian refugees [12]. Living conditions vary deeply between the in-camp and the off-camp Palestinian refugee communities [7]. The literature suggests that the health-related social needs (such as Living Situation, Food, Transportation, Safety, Finance, Employment, Family, Education, Physical Activity, Substance Use, Mental Health, and Disability), and health-related quality of life (including Physical Health, Psychological Health, Social Relationships, and Environmental Health) are likely to vary deeply between the different Palestinian refugee communities according to citizenship status and camp residency [7,9,10,12]. To our knowledge, very few studies compared Palestinian refugees inside and outside camps in Jordan, or compared refugees with and without Jordanian citizenship for their health outcomes, let alone for their health-related social needs and health-related quality of life. Our selection of Gaza camp (housing refugees from the Gaza Strip, mostly without Jordanian citizenship), and Husn camp (housing refugees from the West Bank, mostly with Jordanian citizenship) aims to represent the remaining refugee camps in Jordan.

According to Bronfenbrenner's ecological systems theory (1994), human beings' social and health outcomes are greatly impacted by not only their biological characteristics, but also the ecological systems surrounding them [13]. Therefore, in order to understand an individual's health, health-related quality of life, or health-related social needs, their surrounding multi-layered ecological systems should be understood. In this theory, the immediate environment (microsystem), the connected environments (mesosystems), the indirect environment (exosystem), and the social, political, and cultural environment (macrosystem), in which an individual lives, are related to his/her health outcomes. This study utilizes the ecological model as a theoretical framework, because it can explain the connection between Palestinian refugees' health-related social needs and health-related quality of life and indoor and outdoor environments that vary between inside and outside camps (microsystem and mesosystem), as well as citizenship status (macrosystem) which is greatly likely to influence refugees' access to rights, services, and opportunities [13].

This study aims to assess health-related social needs and health-related quality of life among Palestinian refugees in three settings: Gaza camp, Husn camp, and off-camp Palestinian refugee communities in Amman. by analyzing different factors including mental and physical morbidities, living situations, socioeconomic status and others, and uncovering the main health-related social needs detected to aim for better distribution of healthcare services and eradication of any disparities in the future. The findings of this study can lead to changing the current practices in the healthcare system inside the refugee camps. They can also lead necessary changes in legislation and policies regarding the accessibility and equity of healthcare services for Palestinian refugees inside and outside camps. This study follows the ecological systems theory and hypothesizes that there is a difference in health-related social needs and health-related quality of life between Palestinian refugee populations in the three aforementioned settings, relevant to camp residency and citizenship status.

## 2 Materials & methods

The study follows the Strengthening the Reporting of Observational Studies in Epidemiology cross-sectional reporting guidelines [14].

### 2.1 Study design

The study utilized a descriptive, comparative, cross-sectional design to measure the health-related quality of life and health-related social needs among Palestinian refugees in three settings: Gaza refugee camp, Husn refugee camp, and off-camp Palestinian residents of Amman, Jordan.

### 2.2 Sample & sampling

#### 2.2.1 Sample size

The required sample size was estimated using the Z statistic formula (z = (x-μ)/σ, where x is the raw score, μ is the population mean, and σ is the population standard deviation) given type I error rate (α) of 0.05, type II error rate (β) of 0.2 and an estimated effect size of discrimination of medium level that's equal to 0.4 based on what the literature stated [7]. The equation yielded a minimum required size of 198 to answer the research question and meet the research objectives. An additional 20 % were added to the sample size to account for any missing data resulting in a total sample size of 236 Palestinian refugee participants from the three settings [15,16].

### 2.3 Sampling technique, setting, and inclusion

The study utilized convenience sampling technique to recruit potential participants from Gaza refugee camp, Husn refugee camp, and off-camp neighborhoods in Amman with comparable socioeconomic status. The data was collected between late January and March 2022. A team of data collectors approached the potential participants who needed to be adult (18 years or older), and registered with UNRWA as a Palestinian refugee in the three settings. Data collection took place in markets, streets and other community gatherings. Those who agreed to participate were provided with an information sheet explaining the details of the study and its intended outcomes.

### 2.4 Recruitment and data collection procedure

The research team, which consists of seven medical students, entered the refugee communities after receiving consent from the Department of Palestinian Affairs of Jordan's Ministry of Interior. The purpose of the study, potential risks, and benefits were explained to potential participants. An information sheet was handed to each person who agreed to participate, and a written informed consent form was collected. After that, and the paper-based study questionnaire was filled out by participants, with the assistance of the research team members. Each member of the team was assigned to several people to answer any inquiries they might have regarding the questionnaire, and help illiterate participants using an interviewer-administered data collection approach. Participants were assured that their identities will remain anonymous, and their data will be kept confidential.

### 2.5 Measurements

The study questionnaire was in Arabic and consisted of three parts: (1) socio-demographic characteristics part (age, gender, marital state, health insurance, number of children, education, state of employment, income, current health status, and three 5-point, Likert scale items measuring perceived health discrimination: To what extent does discrimination affect you when it comes to healthcare?’ ‘To what extent are you satisfied with your access to health services?’ And ‘To what extent you able to receive high quality healthcare?‘), (2) the World Health Organization's Brief Quality of Life tool (WHOQOL-BREF) [17], and (3) the Center for Medicare and Medicaid Services' The Accountable Health Communities Health-Related Social Needs Screening Tool (AHC-HRSN) [18]. The study questionnaire was pilot-tested prior to its administration by university-level interviewers selected from different communities.

The second part, the Arabic version of WHOQOL-BREF, is a self-administered questionnaire comprising 26 questions on the individual's perceptions of their health and well-being over the previous two weeks [17]. Responses to questions are built on a 1–5 Likert scale where 1 represents “disagree” or “not at all” and 5 represents “completely agree” or “extremely”. The translated WHOQOL-BREF covers four domains which are: physical health, psychology, social relationships, and environment. The translated Arabic WHOQOL-BREF has considerable reliability and validity indices [19]. The quality of life scores were calculated according to the WHOQOL-BREF guide across the four domains [20].

The third part is the Arabic version of AHC-HRSN screening tool, which was constructed by the Centers for Medicare & Medicaid Services (CMS) [18]. It consists of 26 questions that have been translated using the translation-back translation method. The 26 questions assess five core domains: living situation (having a place of living, worrying about indoor environment, access to water and sanitation), food (worrying about food running out, worrying about not having enough money to buy healthy food), transportation (lack of reliable transportation leading to missing important appointments), utilities (worrying about electric, gas, oil, or water company shutting off services), and safety (being physically hurt by anyone, being insulted, threatened, screamed at, or talked down to); as well as eight additional domains: financial strain (feeling that it is hard to pay for basics), employment (needing help finding or keeping a job), family and community support (feeling lonely, and not getting the needed help in activities of daily living), education (ability speak a language other than Arabic, or help teach one's children or siblings), physical activity (frequently engaging in moderate exercise), substance use (consumption of alcohol, tobacco products, subscription drugs, or illegal drugs), mental health (feeling down, loss of interest, hopelessness, stress, anxiety, or sleep disturbances), and disabilities (experiencing serious difficulty in running errands, visiting doctors, taking decisions, remembering important things because of a physical, mental, or emotional condition) [21]. The ascertainment whether the participants have need in this domain or not was done using the AHC-HRSN scoring guide [22].

### 2.6 Pilot study

A pilot study was conducted on 22 individuals from similar socioeconomic backgrounds of the main study population to test the reliability, usability, clarity, and appropriateness regarding the three sections (socio-demographics, WHOQOL-BREF, and health-related social needs) of the questionnaire and the quality of translation of the third section which assesses the health-related social needs among the studied population. The WHOQOL-BREF section's Cronbach's alpha was measured to be 0.74 while the reliability of the health-related social needs tool was 0.84. The data collected from the pilot study was not included in the major study.

### 2.7 Ethical considerations

This study was conducted in accordance with the ethical guidelines and principles set forth by the Declaration of Helsinki and received approval from the Institutional Review Board (IRB) at the University of Jordan's School of Nursing. (IRB Approval No. PF. 22.1).

Prior to data collection, approval from refugee camp directors and security approval from the Department of Palestinian Affairs was obtained. Every person approached as a potential participant and consent was obtained with a one-page written informed consent information sheet and was reassured that their participation is voluntary, anonymous, and will be kept confidential and that they had the right to choose not to answer any question without any consequences. Data collected were kept private, confidential, secured in a locked cabinet of the principal investigator, and used for research purposes only.

### 2.8 Statistical analysis

All data were exported into Excel sheets before being imported into the Statistical Package for Social Sciences (SPSS) version 25 [23], which was used for data cleaning and statistical analysis. Counts and percentages were used to present categorical variables while mean and standard deviation were used to present continuous variables. Pearson's Chi-square test was used to compare the three settings in categorical variables in terms of health-related social needs. One-Way ANOVA test was used to compare the continuous variables and quality of life scores domains across the three settings. In all inferential statistics, a p-value less than 0.050 was considered statistically significant. Multivariate logistic regression analysis was conducted for the health-related social needs that were significantly different across the three settings to account for confounding variables. Multivariate logistic regression analysis results were presented using Odds Ratio (OR) and their 95 % Confidence Intervals (95 % CI).

## 3 Results

### 3.1 Characteristics of the participants

**Fig. 1:**
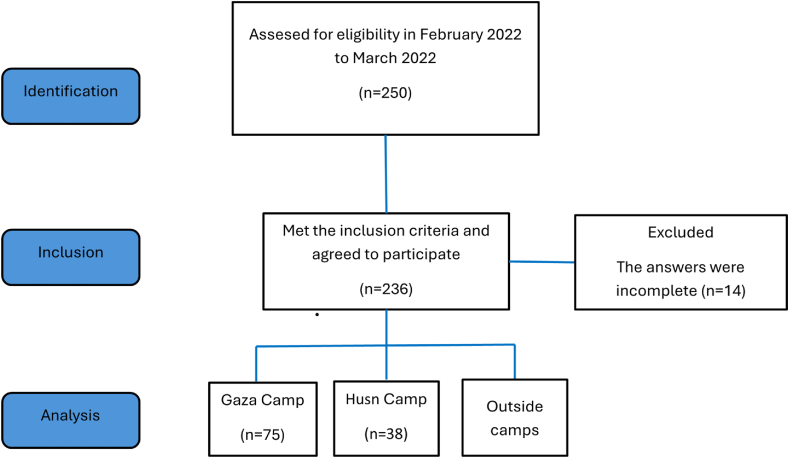
STROBE flow diagram of the study protocol.

The total number of participants in the study was 236 (Fig. 1). In total, 174 (73.7 %) were males and 83 (35.2 %) had no Jordanian citizenship. The mean age of the participants was 39.56 (SD = 14.43) years, and 150 (63.6 %) were married. In addition, 125 (53.0 %) of the participants were employed (mostly in the private sector or freelancers), while the rest were unemployed. The mean income per month was 271.22 ± 173.53 Jordanian Dinars [JD] *(JD 1 ≈ 1.4 US Dollars)* (Table 1).

**Table 1:** The general socio-demographic characteristics of the participants (N = 236).

Characteristic			Frequency	%
Gender	Male		174	73.7
	Female		62	26.3
Jordanian Citizenship	No		83	35.2
	Yes		153	64.8
Marital Status	Single		74	31.4
	Married		150	63.6
	Divorced		7	3.0
	Widowed		5	2.1
Educational Level	Elementary School		31	13.1
	Primary to Intermediate School		70	29.7
	High School		72	30.5
	Diploma		26	11.0
	University or Postgraduate Level		37	15.7
Employment Status	Unemployed		111	47.0
	Employed		125	53.0
Occupation	Government Job		6	2.5
	Private Sector		41	17.4
	Freelancer		26	11.0
	Day Laborer		41	17.4
	Retired		8	3.4
**Characteristic**	**Mean**	**SD**	**Range**	

Age (years)	39.56	14.43	62	
Number of Children	2.78	2.73	15	
Household Members	5.75	2.75	18	
Income (JOD)	271.22	173.53	1200	

SD: Standard Deviation.

JOD: Jordanian Dinar (≈1.4 US Dollars).

### 3.2 The health-related social needs and quality of life among the participants

According to the AHC-HRSN screening tool, 228 (96.6 %) of the participants had a need in the living situation domain, while 179 (75.8 %) had a need in the food domain. Moreover, 190 (80.5 %), 194 (82.2 %) and 202 (85.6 %) of the participants had needs in the safety, financial and family support domains, respectively. 180 (76.3 %) of the participants had need in the employment domain, and 194 (82.2 %) had need in the physical activity domain. All participants had a need in the mental health domain. The WHO-QOL BREF showed that the highest score was in the social relationship domain (mean score = 12.85, SD = 3.40 out of 25), while the lowest score was in the physical health domain (mean score = 11.36, SD = 3.27 out of 25) (Table 2).

**Table 2:** The health-related social needs and quality of life scores among the participants.

Health-Related Social Needs	Outcome		Frequency	Percentage
Living Situation	Yes		228	96.6
Food	Yes		179	75.8
Transportation	Yes		123	52.1
Safety	Yes		190	80.5
Finance	Yes		194	82.2
Employment	Yes		180	76.3
Family	Yes		202	85.6
Education	Yes		24	10.2
Physical Activity	Yes		194	82.2
Substance Use	Yes		164	69.5
Mental Health	Yes		236	100.0
Disability	Yes		131	55.5

**HRQoL Domain Scores**	**Mean**	**SD**	**Range**	

Physical Health Domain	11.36	3.27	16	
Psychological Domain	11.92	2.66	14	
Social Relationships Domain	12.58	3.40	16	
Environment Domain	11.44	3.23	15	

SD: Standard Deviation.

### 3.3 Differences in the demographics between sites of refugees living

Analysis results showed that the percentage of participants who carry Jordanian citizenship was significantly different between the three refugee living settings (13.1 % in Gaza camp, 48.4 % in Husn camp, and 38.6 % outside camps, *p = 0.000*). Education levels differed significantly between the three settings, with higher educational levels in the refugees living outside the camps (*p = 0.040*) as higher percentage of patients have university and postgraduate studies outside camps compared to both Gaza and Husn camps (43.2 % vs. 18.9 % vs. 37.8 %, respectively). The employment percentage was significantly higher outside the camps (27.2 % in Gaza camp, 31.2 % in Husn camps, and 41.6 % outside camps, *p = 0.012*). The mean number of children were significantly higher among participants living in Gaza camp (0.038 (mean = 3.40 ± 2.91 for Gaza Camp, 2.20 ± 2.34 for Husn camp, and 2.78 ± 2.85 for outside camp, *p = 0.038*). Number of and household members also varied significantly (P = 0mean = 6.69 ± 2.86 for Gaza camp, 5.45 ± 2.40 for Husn camp, and5.17 ± 2.79 for outside camps, *p = 0.001*). The participants’ monthly income was significantly higher among refugees living outside the camps (P = 0mean = 205.28 ± 147.75 for Gaza camp, 263.76 ± 164.95 for Husn camp, and 342.55 ± 180.27 for outside camps, *p = 0.000*) (Table 3).

**Table 3:** Differences in the socio-demographic characteristics between sites of refugees living.

Variable		Gaza Camp (n = 75)	Husn Camp (n = 83)	Outside Camps (n = 78)	*p*
Gender	Male	59 (33.9)	63 (36.2)	52 (29.9)	*0.206*
	Female	16 (25.8)	20 (32.3)	26 (41.9)	
Citizenship	Yes	20 (13.1)	74 (48.4)	59 (38.6)	*0.000*^a^
	No	55 (66.3)	9 (10.8)	19 (22.9)	
Marital Status	Single	25 (33.8)	24 (32.4)	25 (33.8)	*0.460*
	Married	45 (30.0)	56 (37.3)	49 (32.7)	
	Divorced	2 (28.6)	1 (14.3)	4 (57.1)	
	Widowed	3 (60.0)	2 (40.0)	0 (0.0)	
Education Level	Elementary School	12 (38.7)	15 (48.4)	4 (12.9)	*0.040*^a^
	Primary to Intermediate School	28 (40.0)	25 (35.7)	17 (24.3)	
	High School	19 (26.4)	22 (30.6)	31 (43.1)	
	Diploma	9 (34.6)	7 (26.9)	10 (38.5)	
	University to postgraduate	7 (18.9)	14 (37.8)	16 (43.2)	
Employment Status	Unemployed	41 (36.9)	44 (39.6)	26 (23.4)	*0.012*^a^
	Employed	34 (27.2)	39 (31.2)	52 (41.6)	
Age		40.03 ± 16.04	38.73 ± 13.15	40.00 ± 14.23	*0.083*
Number of Children		3.40 ± 2.91	2.20 ± 2.34	2.78 ± 2.85	*0.023*^a^
Household Members		6.69 ± 2.86	5.45 ± 2.40	5.17 ± 2.79	*0.001*^a^
Monthly Income		205.28 ± 147.75	263.76 ± 164.95	342.55 ± 180.27	*0.000*^a^
Physical Health Domain		10.93 ± 3.36	111.70 ± 3.23	11.41 ± 3.23	*0.331*
Psychological Domain		11.47 ± 2.90	12.09 ± 2.49	12.18 ± 2.57	*0.197*
Social Relationships Domain		12.32 ± 3.70	13.08 ± 2.91	12.31 ± 3.57	*0.260*
Environment Domain		11.03 ± 3.73	11.36 ± 2.69	11.90 ± 3.24	*0.241*

a Statistically significant; p < 0.05.

### 3.4 Differences in health-related social needs and quality of life between sites of refugees living

**Fig. 2:**
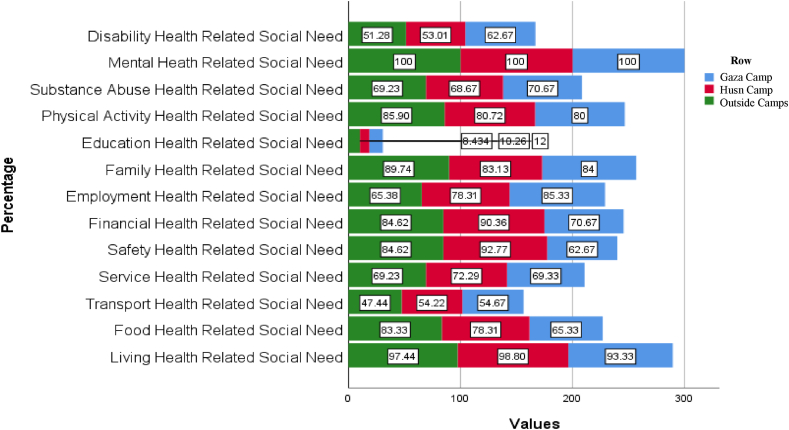
Differences in health-related social needs and quality of life between sites of refugees living.

Regarding the differences in health-related social needs and quality of life between the three living sites (Fig. 2), higher percentage of participants had needs in the food domain in the Husn camp and participants living outside camps (36.3 % for each) compared to Gaza camp (27.4 %) (*p = 0.027*). A higher percentage of participants had needs in the safety domain in Husn camp compared to the two other settings (*p = 0.000*; 92.77 % for Husn camp vs 84.62 % for Gaza camp and 62.67 % for outside camp). Moreover, a higher percentage of participants in Husn camp (38.7 %) had needs in the financial domain compared to Gaza camp (27.3 %) (*p = 0.004*). A higher percentage of need in the employment domain among participants living in Gaza (35.6 %) and Husn (36.1 %) camps compared to the participants living outside camps (*p = 0.013*). No differences in any of the quality of life domains existed between the participants living in the three settings (Table 3).

### 3.5 The association between health-related social needs and sites of refugees living

Monthly income and number of household members were the only factors associated with the need in the food domain (OR = 1.004; 95%CI: 1.001 to 1.007, OR = 0.843; 95%CI: 0.740 to 0.961). Regarding the safety needs, living in both Gaza and Husn camps was significantly associated with higher odds of safety need compared to living outside camps (OR = 4.27; 95%CI: 1.541 to 11.76, OR = 2.936; 95%CI: 1.942 to 9.147). Living in Gaza and Husn camps was significantly associated with higher odds of financial need compared to living outside camps (OR = 6.135; 95%CI: 2.062 to 18.181, OR = 3.932; 95%CI: 1.765 to 8.210). In addition, not having citizenship was significantly associated with higher odds of financial needs (OR = 2.817; 95%CI: 1.109 to 7.154). Moreover, living in Gaza and Husn camps was significantly associated with higher odds of employment need compared to living outside camps (OR = 2.349; 95%CI: 1.877 to 6.291, OR = 2.406; 95%CI: 1.050 to 5.514) (Table 4).

**Table 4:** Multivariate regression analysis for the association between health-related social needs and sites of refugees living.

Variable		Food OR (95%CI)	Safety OR (95%CI)	Financial OR (95%CI)	Employment OR (95%CI)
Location	Gaza Camp	0.951 (0.366–2.476)	4.27^a^ (1.541–11.76)	6.135^a^ (2.062–18.181)	2.349^a^ (1.877–6.291)
	Husn Camp	0.959 (0.381–2.419)	2.936^a^ (1.942–9.147)	3.932^a^ (1.765–8.210)	2.406^a^ (1.050–5.514)
	Outside Camps	Reference	Reference	Reference	Reference
Citizenship	No	0.781 (0.355–1.717)	2.251 (0.930–5.449)	2.817^a^ (1.109–7.154)	1.504 (0.652–3.471)
Education Level	Elementary School	0.249 (0.044–1.419)	0.718 (0.162–3.181)	1.114 (0.270–4.606)	0.288 (0.072–1.155)
	Intermediate School	0.274 (0.05–1.405)	1.185 (0.335–4.197)	2.450 (0.724–8.290)	0.571 (0.173–1.892)
	High School	0.376 (0.074–1.910)	1.571 (0.459–5.376)	1.784 (0.598–5.319)	0.786 (0.258–2.395)
	Diploma	0.179 (0.031–1.048)	2.855 (0.510–15.987)	1.641 (0.416–6.477)	0.457 (0.126–1.661)
	University to postgraduate	Reference	Reference	Reference	Reference
Employed	No	0.453 (0.341–1.612)	0.895 (0.531–1.894)	1.311 (0.781–4.390)	1.891^a^ (1.100–5.912)
Number of Children		0.977 (0.858–1.113)	0.998 (0.861–1.158)	0.996 (0.854–1.61)	0.931 (0.814–1.064)
Household Members		0.843^a^ (0.740–0.961)	0.934 (0.815–1.071)	1.082 (0.930–1.258)	1.042 (0.905–1.200)
Monthly Income		1.004^a^ (1.001–1.007)	1.002 (0.999–1.004)	0.999 (0.997–1.001)	0.997^a^ (0.995–0.999)

a Statistically significant; 95 %, and95 % CI does not cross 1.00.

## 4 Discussion

Refugee camps are tucked away in Jordan's landscape and serve as inspiration for resilience by providing shelter and hope to people who have been uprooted by war. These camps tell stories of perseverance and survival in the face of hardship, portraying the unwavering essence of the human experience. Nevertheless, a significant number of these refugee camps are unable to maintain a conducive environment concerning the physical, psychological, and socio-economic aspects of life. According to the study's results, a substantial portion of participants showed health-related social needs in various domains. These included the living situation, food, safety, financial support, and family support domains. Employment and physical activity domains also showed significant needs. Remarkably, all participants required assistance in the mental health domain.

The analysis revealed significant differences among the three refugee living sites. Citizenship status, education levels, employment rates, average number of children and household members, as well as monthly income, all exhibited significant variations across these sites. Moreover, the study found that participants in Husn and Gaza camps had higher food, safety, financial, and employment needs compared to participants living outside camps.

Regarding educational backgrounds, Habib et al. reported that 14 % of Palestinian refugees in Lebanon had received no formal education, with 29 % having completed elementary school and 38 % reaching intermediate school levels. The remaining participants included 10 % with secondary school diplomas, 3 % who had undergone vocational training, and 6 % who pursued higher education [24]. In contrast, this study's showed that 13.1 % completed elementary school, 29.7 % achieved intermediate school education, 30.5 % attained high school qualifications, 11 % earned diplomas, and 15.7 % continued their education at the university or postgraduate level. While there are similarities in the focus on health among Palestinian refugees, there are variations in the specific health conditions studied and differences in the demographic characteristics of the participants between the two research efforts.

A significant 73 % of the Palestinian population residing in the Gaza camp did not have Jordanian citizenship. This can be attributed to the fact that most residents of Gaza camp were originally displaced from the Gaza Strip after the 1967 s Arab-Israeli war, and were issued temporary passports [12]. Palestinians who lived outside the camps exhibited higher income levels when compared to those residing in both Gaza and Husn camps, which is consistent with the findings of Alduraidi and Waters (2017) who reported higher levels of poverty inside Palestinian refugee camps. The combination of living within refugee camps and lacking Jordanian citizenship was linked to greater financial needs, aligning with findings from previous data outlined in the FAFO Report of 2014 [25]. Existing literature has indicated that refugees based in camps are notably more likely to possess a lower per capita income than those living outside of camps [7]. Camp-based refugees are also more prone to living below the national poverty line, resulting in difficulties in meeting fundamental daily necessities, especially in the wake of *the coronavirus disease 2019 (COVID-19*) pandemic [26]. Primary sources of household income in refugees primarily stem from waged labor, loans, and the sale of household belongings and assets [26]. These temporary work opportunities do not provide stable incomes, making households vulnerable to economic hardship. A smaller percentage rely on assistance from United Nations agencies (UNICEF) and non-governmental organizations (NGOs) [27].

In terms of household size, Gaza camp had significantly higher crowdedness with a mean size of 6.7 members per household. Camp residents are more likely to suffer from household crowding than outside camp refugees [27]. In 2011, the mean household size in Palestinian refugee camps was 5.1 with Gaza camp bearing the largest mean household size of 5.8 with approximately 20 % of households having twice the average household size of other Palestinian refugee camps based in Jordan [25]. The UNICEF report on Gaza Camp showed that in 2021 the mean household size was 7.4 members with 71 % of households having 6 to 10 members [27]. AlNsour and Meaton found that the mean household size of refugees living in Al-Baqaa camp of Amman was 6.4 [28]. Although there has been a rise in nuclear households in expense to extended households, the study reported a high number of extended households in Gaza camp which may justify the findings [25]. Household crowding has been linked to multiple morbidities including mental health issues and communicable diseases [29]. Additional infrastructure plans are required to reduce household overcrowding and improve housing conditions.

Educational attainment among camp-based refugees was lower. This finding aligns with existing studies conducted among Palestinian refugees [12,14,24,30,31]. This finding might be attributed to the cost of university education in Jordan, where a family with low income is probably unable to support its young members’ university education financially. Furthermore, the cost of university education for non-citizen Palestinian refugees in Jordanian public and private academic institutions is often higher, because they are considered international students. Additionally, it could be associated with the relatively higher socio-economic status of individuals living in Amman in comparison to other regions. Palestinian refugees lacking citizenship are unable to access public schools, leading them to attend UNRWA agency schools or private schools that may be financially challenging to afford. Many of the school buildings provided by UNRWA lack essential facilities like elevators or ramps, thus failing to address the needs of individuals with disabilities [32]. Al-Najjar et al.'s study on safety concerns for school students in Gaza refugee camp, such as the low walls of schools and unprotected class windows, could potentially contribute to the high dropout rates observed; as the authors suggest [33]. To address such safety concerns, responsible authorities should aim to establish a safe school environment and provide free transportation services, especially for children. In addition, governments should build programs that investigate the causes of school dropouts and address children who drop out through social workers. Agencies should aim to increase the percentage of scholarships offered to camp residents in both public and private universities.

Palestinians living outside the camps exhibited higher employment rates. Jordan has experienced a 23.3 % surge in unemployment following the COVID-19 pandemic, and this issue is expected to be even more severe among refugees inside camps [34]; where job opportunities are scarcer than outside camps. Many Palestinian refugees, who predominantly depended on wage labor, lost their jobs during nationwide lockdowns and social distancing mandates, forcing them to rely on their savings in the absence of a fixed income [30]. Previous data showed that Husn camp suffered from high unemployment percentages [12]. Gaza camp also showed similar trends, with over half of its residents reporting unemployment [27]. Residents of Gaza camp had the least percentage in working within the governmental sector [35]. These findings may stem from the restrictions imposed on refugee camp residents' mobility and limited employment opportunities within the private and public sector due to the temporary residency status of Gaza camp residents [36]. The constrained opportunities drive many residents of Gaza camp to seek illegal jobs, driving them to job instability, lower wages and legal consequences [37]. To reduce unemployment rates, it is necessary to ease restrictions on certain professions. This will help integrate refugee residents, especially those without citizenship, in both public and private sectors. Further support should be provided with local home businesses, i.e. by assisting them with raw materials. Providing career guidance on in-demand job opportunities for youth residing in camps may also help in reducing unemployment in the future generations. A collaboration between United Nations Educational, Scientific and Cultural Organization (UNESCO) and the private sector can help in establishing training programs for fresh graduates residing in refugee camps. Further studies are needed to assess the possible factors that increase unemployment, such as transportation access and discrimination, as research in this area is limited in Jordan and the Middle East.

While the data underscored that Palestinians residing in both Husn camp and outside camps exhibited high vulnerability to food insecurity and insufficiency, subsequent regression analysis revealed that monthly participant income and household size were the key determinants of food security and sufficiency. Location was no longer significant in the regression after accounting for potential confounders. This finding aligns with prior scholarly works which have consistently suggested a pronounced correlation between impoverished households and heightened susceptibility to severe food insecurity, particularly within refugee communities [38,39]. Extant literature from various countries has concurred on the influential role of household size in shaping levels of food insecurity [40]. However, Ghattas et al. studying Palestinian refugees in Lebanon has not attributed the same significance to household size in its impact on food insecurity [38]. This could be attributed to the circumstance that Jordan's food commodity imports greatly surpass its exports, resulting in the country facing a food supply deficit [41]. Additionally, factors like drought and shifts in climate patterns also contribute to this situation [42]. Food insecurity may have been further exacerbated by the loss of jobs during the pandemic [43]. To comprehensively address the implications of the pandemic on food insecurity and food insufficiency, particularly within vulnerable demographics, further projects are to be conducted on a local and regional level to assess the observed trends. These inquiries should delve into the aftermath of the pandemic and its potential amplification of food insecurity, alongside its cascading effects on exacerbating health-related issues [38,44]. Furthermore, this exigency highlights the necessity for heightened efforts in instituting food assistance programs within refugee camps. Such initiatives align with the objectives of the 2nd Sustainable Development Goal, which centers on eradicating hunger and malnutrition.

Residents of the camps experienced higher levels of safety concerns within their families, social circles, and overall environment compared to those living outside the camps. According to UNRWA's 2013 report, nearly all individuals, both inside and outside the camps, reported feeling secure within their homes, though most Palestinian refugees within camps felt unsafe in their residential areas compared to those outside the camps [12]. Currently, research examining the safety requirements of refugees globally, particularly within the Middle East, is insufficient. Further investigations are necessary to delve into the variations in safety needs across different age and gender groups, to appropriately address these needs.

The mental health need existed in 100 % of participants. This is consistent with the findings of previous studies, such as Alduraidi and Waters’ 2018 study which found that a great percentage of Palestinian refugees in all places of residence had moderate to severe depressive symptoms [45]. Also, in their 2017 study, Alduraidi and Waters [7] reported that psychological health scores of Palestinian refugees both inside and outside camps in Jordan were equally low with no significant differences among the two populations.

Although previous studies found differences in physical health, and environmental domains of health-related quality of life where in-camp refugees fared significantly worse than their outside-camp counterparts [7], no variations were observed in the current study. This may be attributed to the fact that after the COVID-19 pandemic, the physical health characteristics have deteriorated both inside and outside camps. Furthermore, Palestinian refugees in the three settings where data was collected seem to suffer from common hardships despite differences in some particular characteristics. The environmental aspect of health-related quality of life outside camps seems to be deteriorating since the COVID-19 pandemic as well. Further research is however needed to better understand quality of life differences in different Palestinian refugee communities in Jordan using mixed-method comparative designs.

### 4.1 Limitations

The study's cross-sectional design did not enable us to investigate causal linkages and made the progression of events over time less clear. However, the study established significant differences that offer valuable insights into how assistance policies and programs could mitigate the health-related social needs of camp residents, particularly during critical circumstances, such as pandemics. Due to strict security regulations within the refugee camps, the study employed a non-probability, convenience sampling technique to recruit participants. Nonetheless, the demographic characteristics of the sample closely resembled those of the official Palestinian refugee population in Jordan [12,27,46]. Predominantly male survey respondents were recruited, primarily because they were more accessible in public spaces like streets and markets, yet this is unlikely to affect the study's results since male representation was not different between the three settings. The utilization of self-reported data introduces a subjective element that could potentially be influenced by recall bias. Although the authors accounted for confounding factors through regression analysis, the possibility of confounding bias cannot be fully excluded.

### 4.2 Policy implications

Recently, major global donors of UNRWA have been cutting off of cutting down their donations to the organization because of political or economic reasons. This decrease in funding threatens the livelihood of millions of Palestinian refugees in Jordan and elsewhere, who rely on UNRWA for basic everyday services like healthcare and education. The global community should reverse this trend and increase its funding of UNRWA to help meet the many needs of Palestinian refugees. Also, the government of Jordan should consider either granting full citizenship to Palestinian refugees of Gazan origins, who were born and raised in Jordan; or change the laws to improve their access to jobs, and their ability to own properties and start businesses, in order to meet their needs and improve their livelihoods.

Health-related social needs were found in all Palestinian refugee communities, indicating that the policy changes are necessary to address these needs. Expanding health insurance coverage to include Palestinian refugees inside and outside camps, regardless of their citizenship status, is crucial to meeting their physical and mental health needs. Campaigns for combating substance use are also necessary within Palestinian refugee communities. Labor policies should be changed, and effective partnerships between public and private sectors are needed so Palestinian refugees with or without citizenship are able to find well-paying jobs to save themselves and their families from the damaging consequences of poverty and unemployment. For this purpose, educational and vocational training programs, capacity building efforts, and microfinance campaigns are crucially needed within Palestinian refugee communities. Food security within Palestinian refugee communities should be prioritized in Jordanian government strategic plans, through adopting appropriate programs, such as distributing food stamps, or food discount coupons for refugee families. In addition, governmental organizations should run necessary checks to monitor food quality, prices, and availability inside Palestinian refugee camps. Transportation networks in Palestinian refugee communities should be expanded and enhanced to increase refugees’ ability to move freely, quickly, and cheaply, which can diversify their options for employment, education, and healthcare service utilization.

The Jordanian government should also subsidize the prices of utilities, such as electric, oil, and sewer services for Palestinian refugee household, especially inside camps. The Jordanian government with UNRWA's and other relevant organizations' assistance should reform the safety and security status within Palestinian refugee communities, by strengthening low-enforcement role, funding behavioral rehabilitation services for individuals with criminal history, and creating effective and confidential counseling services for victims of homicide or domestic violence. Loans, grants, microfinance, and financial aid programs with no or low interests should be made available to Palestinian refugee families and startups, facilitating their success in business to improve their economic status. The Jordanian government with the help of UNRWA, private sector, and other organizations should invest in industrial or service projects in or near Palestinian refugee communities to create new job opportunities for refugee men and women of all ages, to help resolve their unemployment need. Furthermore, a decent minimum wage should be imposed for refugee employees, along with decent health insurance and social security benefits.

Social welfare programs should be created in Palestinian refugee communities, targeting those with no or minimal family or social support, such as widows, orphans, and single mothers. Also, social clubs and support groups should be established inside camps to help refugees with common interests network and share resources and experiences. Funding educational facilities that serve Palestinian refugees, including UNRWA's schools and colleges, should be a priority in the Jordanian strategic planning. Public, private, and philanthropic sectors should also expand and increase educational grants and loans directed to Palestinian refugee youth, especially those without Jordanian citizenship. Universities should also consider offering waivers or discounts for Palestinian refugee students, particularly those who live in camps, those who do not have Jordanian citizenship, and those who have uneducated or poorly educated parents. Adequate and affordable recreational and physical activity facilities should be established in or near Palestinian refugee communities, particularly those inside camps, along with awareness spreading campaigns encouraging refugees of all ages and of both genders to adopt active lifestyles. Mental health and psychosocial support services should be widely and effectively available to Palestinian refugees inside and outside camps to meet the alarming 100 % mental health need that this study found. These services should include pharmaceutical and non-pharmaceutical treatments of mental health problems. Equal access to services and job opportunities should be guaranteed for people with disabilities in the Palestinian refugee communities by reforming the existing relevant polices.

## 5 Conclusion

Jordan, a lower middle-income country hosts over two million UNRWA-registered Palestinian refugees, most of whom reside outside camps. Palestinian refugees who still reside in the country's 13 refugee camps suffer from a clear concentration of poverty and unemployment, among many other problems. While most Palestinian refugees in Jordan have full Jordanian citizenship, those who originate from the occupied Gaza Strip and whose ancestors fled to Jordan in the late 1960's do not have this right. The Gaza camp in northern Jordan houses a majority of Gazan Palestinian refugees with no Jordanian citizenship, who seem to suffer the most from harsh living conditions, limited job opportunities, and very low socio-economic status.

This study assessed health-related social needs and health-related quality of life in three communities of Palestinian refugees; camp refugees without citizenship (Gaza camp), camp refugees with citizenship (Husn camp), and off-camp refugees with citizenship (Amman). The results showed that all three communities have alarming health-related social needs and have generally low health-related quality of life. The odds of having safety needs are significantly about four times greater in Gaza camp, and significantly about three times greater in Husn camp than outside camps. The odds of financial needs are significantly about six times greater in Gaza camp, and significantly about four times greater in Husn camp than outside camps. The odds of employment needs are significantly over twice greater in Gaza camp and in Husn Camp than outside camp. Furthermore, the odds of financial needs are nearly three times greater among Palestinian refugees without Jordanian citizenship than among those with Jordanian citizenship.

Major countries and international organizations should help the Jordanian government respond to the alarming health-related social needs of Palestinian refugees by directly donating resources to the Jordanian government, or indirectly by increasing the funds for UNRWA. Special measures should be taken to improve the quality of life and meet the social needs of refugees inside camps, particularly those without citizenship. Laws and policies need to be changed to respond to the social needs in all Palestinian refugee communities in Jordan, but with a special emphasis on safety, financial, and employment needs inside camps, and improving the livelihood of the Gaza camp inhabitants namely. Further research is needed to better understand the lived experiences of Palestinian refugees in Jordan, especially after the damage done to them during the COVID-19 pandemic.

## CRediT authorship contribution statement

**Hamza Alduraidi:** Writing – review & editing, Supervision, Project administration, Methodology, Formal analysis, Conceptualization. **Rahaf Issam Abu Zayid:** Writing – review & editing, Writing – original draft, Project administration, Methodology, Investigation, Data curation, Conceptualization. **Noor Mahmoud Jaber:** Writing – original draft, Methodology, Investigation, Conceptualization. **Bassam Saleh Hijazi:** Writing – original draft, Methodology, Investigation, Data curation. **Sa'ed Radwan Khamis:** Writing – original draft, Methodology, Investigation. **Hasan Fahed Awad:** Writing – original draft, Methodology, Investigation. **Marah Al-Khateeb:** Writing – original draft, Investigation. **Saif alnour Mahmoud Aldhirat:** Writing – original draft, Investigation. **Ahmad Amjed Toubasi:** Writing – review & editing, Writing – original draft, Visualization, Formal analysis.

## Data availability

Data are available upon reasonable request.

## Ethics and consent statement

All study participants provided written informed consent to participate in the study and for their data to be published. The study was reviewed and approved by The Institutional Review Board (IRB) at the University of Jordan's School of Nursing with the reference number (PF. 22.1) dated 23^/^Jan/2022.

## Declaration of competing interest

The authors declare that they have no known competing financial interests or personal relationships that could have appeared to influence the work reported in this paper.
